# Excusing Psychedelics and Accommodating Psychedelics

**DOI:** 10.1080/15265161.2024.2433441

**Published:** 2025-01-13

**Authors:** Edward Jacobs

**Affiliations:** University of Oxford

Cheung et al. (2025) rightly argue that psychedelics should be subject to ethical and evidentiary standards consistent with clinical medicine at large, rather than exceptionalism based on their uniqueness. From near-weekly conversations with the authors on a broad range of shared interests within psychedelic research, and our collaboration on the recent Hopkins-Oxford Psychedelic Ethics (HOPE) Consensus Statement (Jacobs et al. 2024a), I am hopeful that our positions on exceptionalism align more than they diverge. Whether the divergence below reflects a difference in substance or simply in emphasis, I leave to the reader’s judgment.

With my recent article on the inadequacy of informed consent for psychedelic therapies (P-AT) (Jacobs 2023) mentioned by both of the “exceptionalism” articles in this special issue, I feel it important to underline that *business-as-usual* and *reinventing the bioethical wheel* are not the only options for responding to P-AT. We want to maintain our ethical and epistemic standards when thinking about psychedelics. But that doesn’t necessarily mean we can go about meeting these standards in the same way.

In the main, Cheung et al. reject the idea that “psychedelics or psychedelic treatments are so radically different from anything else in psychiatry or medicine that they should be treated in an *exceptional* manner” (19). Psychedelics do have a knack for evoking strong emotions, whether they are taken in a clinical trial, at a music festival, or are simply being talked about on Twitter or LinkedIn. Because of this, Cheung et al. are right to identify and warn against both the “positive” flavor of psychedelic exceptionalism (“psychedelics are so profound, beneficial, important and so on, that ‘the usual rules shouldn’t apply’”(23)), and the “negative” (“[there is] a need to subject psychedelics to more stringent ethical or research requirements than other medical interventions” (23)).

What this analysis doesn’t capture so well is the sentiment that, yes, “consistent ethical rules and evidentiary standards should be applied across all relevant areas of clinical medicine” (16), but following those rules and meeting those standards might, sometimes, require a different approach. In my reading of the literature, I don’t encounter calls for lowered standards. Rather, what I see are arguments that different approaches may be needed to meet the same ethical requirements we hold across medicine. Where Cheung et al. see calls for *different rules* for psychedelics, I tend to see appeals for *different tools*. Those arguing to incorporate different trial designs (including pragmatic and observational cohort trials) alongside RCTs when evaluating psychedelic therapies may be doing so because they consider psychedelics too profound, beneficial, etc, to be subject to proper scrutiny. A more prosaic explanation is that these changes are appropriate because psychedelics are *annoying and recalcitrant* and don’t fit our preferred tools very well. To the extent that double-blind, randomized, placebo-controlled trials work well for drug evaluation, it’s because they are designed to preserve patient and researcher ambiguity about whether an active dose has been taken, and to discount extrapharmacological influences on outcome. To look for alternative investigational approaches for an intervention which has hard-to-blind subjective effects, and that is presumed to depend on extrapharmacological factors (the much discussed “set and setting”), seems more to me like seeking to *accommodate* psychedelics, rather than *excuse* them from rigorous scrutiny (Villiger in press).

This is on a par with at least some of the psychedelic *ethical* exceptionalism that is discussed. I previously argued (Jacobs 2023) that, because of its potentially epistemically and personally transformative nature, informed consent may not be the best tool for legitimize P-AT (“along with any other medical interventions which involve transformative experience” (p.7)). Given the centrality of informed consent to medical ethics, I can see why casting doubt on it may seem radical. But it is already accepted that informed consent is not the right tool for every job in medicine. There is little that a three-year-old can provide informed consent for. Likewise the unconscious trauma victim who is rapidly bleeding out. Yet pediatric and emergency medicine continue nonetheless, using alternative tools—proxy consent, implied consent—where informed consent is not an option. These tools neither amount to a carte blanche for any kind of medical action, nor do they constitute exceptionalism. Instead, they underline that our ethical principles in medicine are not absolute, but are balanced against each other and shaped by the situations in which they’re applied: calibrating our ethical approaches according to specific contexts is a markedly unexceptional aspect of medicine.

More broadly, what might be viewed as psychedelic ethical exceptionalism might, at least sometimes, be seen instead as a process of reflective equilibrium to find a balance between innovation and continuity—seeing psychedelic treatments as a challenge and an invitation to stretch and adapt our existing frameworks without breaking them, and to find ways of accommodating new realities within established principles.

Such a stance broadly coheres with the behavior of the FDA of late. In their recent draft guidance for researchers investigating psychedelic interventions (FDA 2023), they emphasize that psychedelics are “subject to the same regulations and same evidentiary standards for approval as other drug development programs.” This statement is clearly in line with the rejection of exceptionalism envisioned by Cheung et al., but its placement within a document specifically discussing special considerations for research in psychedelics suggests that the atypicality of psychedelics demands some special consideration to meet those standards.

That psychedelic therapy—a hybrid pharmacological-psychotherapeutic intervention—sits uncomfortably within the frameworks for drug testing is not a novel observation. But rather than relitigating well-trodden debates about how best to count or discount the role of extrapharmacological factors on outcomes, I’ll point instead to the limitations of psychedelic adverse event (AE) reporting. Simply put, psychedelic research does not do AE reporting well (Hinkle et al. 2024). The literature’s approach is heterogeneous and unsystematic, leaving us with imprecise estimates of the true risks involved.

But the wide-ranging effects of psychedelic interventions plausibly demand new tools for reporting, assessing, and managing AEs too. Writing in 2019, sociologist Tehseen Noorani noted that “[psychedelic] clinical trial data tend to ignore … new problems and riddles that emerge from participant experiences when these are unrelated to the clinical targets the trials were designed to treat.” (Noorani 2019, 36). This claim aligns with my own interviews with psychedelic trial participants about the challenges they faced in the months and years that followed their treatment. Thorny and distressing problems related to personal identity, arrested integration, and new insights on important social relationships may not be unique to psychedelics, but they are not uncommon (Evans et al. 2023). The forms of distress and struggle that arose following P-AT in my interview cohort might be well-captured as “unwanted events” under some psychotherapeutic frameworks (Linden 2013), but were effectively invisible to the adverse event recording in their respective trials. It is in part because of these struggles that I have elsewhere argued for expanded post-trial access and care in psychedelic research that substantially exceeds that typically found in biomedical research (Jacobs et al. 2024b). This, too, is not about psychedelic exceptionalism. Rather, I suggest that the same ethical standards we should apply to all research may necessitate different demands for interventions with distinctive (if not unique) features and outcomes.

Providing prospective patients with a comprehensive account of the potential risks and outcomes of P-AT is surely of ethical concern, and developing new tools and frameworks to do so is warranted. Palitsky et al. (2024) proposed framework allows space to track Spiritual, Existential, Religious and Theological outcomes, while Calder and Hasler’s Swiss Psychedelic Side Effects Inventory permits variation in the subjective valence attributed to side-effects. This much is important when some outcomes of P-AT that might be seen as untoward or unwelcome are actually endorsed, on the whole, as positive—as, e.g., crying and feelings of anxiety (Calder and Hasler 2024).

Looking for and developing different tools for psychedelic research isn’t (always) exceptionalism; rather, it can be about developing innovative means to uphold—not exempt psychedelics from—established standards in a new environment. Cheung et al. remind us that we may not need to reinvent the bioethical wheel. Agreed! But let’s not preclude ourselves from developing bioethical differential steering. Introducing differential steering allowed vehicles to adapt to varying road conditions without fundamentally altering the core working of the wheel. So too can we adapt our bioethical frameworks to be fine-tuned to address features of psychedelics which, individually or in aggregate, make them a less comfy fit with our usual way of doing things. Smith and Sisti’s (2020) enhanced consent, for example, is not reinventing the wheel but adjusting how it works. It optimizes consent for a new context, rather than reinventing it anew. And, *per* Cheung et al., if it has applications elsewhere in medicine, so much the better.
